# Caloric Restriction *per se* Rather Than Dietary Macronutrient Distribution Plays a Primary Role in Metabolic Health and Body Composition Improvements in Obese Mice

**DOI:** 10.3390/nu13093004

**Published:** 2021-08-28

**Authors:** Petras Minderis, Andrej Fokin, Mantas Dirmontas, Mindaugas Kvedaras, Aivaras Ratkevicius

**Affiliations:** 1 Institute of Sport Science and Innovations, Lithuanian Sports University, 44221 Kaunas, Lithuania; andrej.fokin@lsu.lt (A.F.); mindaugas.kvedaras@lsu.lt (M.K.); aivaras.ratkevicius@lsu.lt (A.R.); 2 Department of Health Promotion and Rehabilitation, Lithuanian Sports University, 44221 Kaunas, Lithuania; mantas.dirmontas@stud.lsu.lt

**Keywords:** low-carbohydrate, low-fat, high-protein, diets, weight loss

## Abstract

Caloric restriction (CR) is of key importance in combating obesity and its associated diseases. We aimed to examine effects of dietary macronutrient distribution on weight loss and metabolic health in obese mice exposed to CR. Male C57BL/6J mice underwent diet-induced obesity for 18 weeks. Thereafter mice were exposed to a 6-week CR for up to 40% on either low-fat diet (LFD; 20, 60, 20% kcal from protein, carbohydrate, fat), low-carb diet (LCD; 20, 20, 60% kcal, respectively) or high-pro diet (HPD; 35, 35, 30% kcal, respectively) (*n* = 16 each). Ten mice on the obesogenic diet served as age-matched controls. Body composition was evaluated by tissue dissections. Glucose tolerance, bloods lipids and energy metabolism were measured. CR-induced weight loss was similar for LFD and LCD while HPD was associated with a greater weight loss than LCD. The diet groups did not differ from obese controls in hindlimb muscle mass, but showed a substantial decrease in body fat without differences between them. Glucose tolerance and blood total cholesterol were weight-loss dependent and mostly improved in LFD and HPD groups during CR. Blood triacylglycerol was lowered only in LCD group compared to obese controls. Thus, CR rather than macronutrient distribution in the diet plays the major role for improvements in body composition and glucose control in obese mice. Low-carbohydrate-high-fat diet more successfully reduces triacylglycerol but not cholesterol levels compared to isocaloric high-carbohydrate-low-fat weight loss diets.

## 1. Introduction

Prevalence of overweight and obesity continues to increase worldwide and it is becoming a major factor contributing to a higher all-cause mortality [1]. There is now convincing evidence that accumulation of excess body fat is associated with susceptibility to the whole range of chronic diseases and syndromes and one of the primary goals of public health policy is to employ successful strategies to reduce levels of obesity in many countries around the world [2].

Accretion of body fat is attributed to energy imbalance which can be modified with a diet and physical activity. It seems that adjustments of diet are of major importance for long-term success in weight management [3]. Reduction in food intake or caloric restriction (CR) is a prerequisite for weight loss, but macronutrient composition of the diet is also suggested to be important [4]. Overfeeding studies suggest that dietary protein has a smaller obesogenic effect and leads to greater increase in lean body mass compared to carbohydrates or fat [5]. It appears that dietary protein has a greater effect on satiety [6] and dietary-induced thermogenesis [7] which both help to maintain energy balance. Increase in protein consumption might reduce CR-induced decrease in the basal metabolic rate and thus counteract weight regain during weight maintenance phase [8,9]. According to Hall [10], fractional calories from protein are decreasing in the US due to the greater increases in carbohydrate and fat content of the diets, and this development might contribute to the obesity epidemics. Protein leverage theory proposes that dietary protein is a macronutrient of high priority and individuals will eat until protein needs have been met, regardless of calorie intake [11]. This theory has been partially supported by rodent studies where energy intake and fat storage were lower when mice were fed high-protein compared to low-protein foods [12,13].

In contrast to dietary protein, there is a significant amount of controversy regarding effects of carbohydrate and fat in the diets for weight management and health [14,15]. This is probably due to the methodological problems in assessment of food intake, standardization of nutrient content in diets and heterogeneity of the volunteers in human nutrition studies [16]. Thus, there is a need for the rigorously controlled feeding studies examining the importance of macronutrient composition of diets for health [17].

Studies of obesity-prone C57BL/6J mouse strain have been useful in dissecting physiological mechanisms of obesity and diabetes [18,19]. Mouse model offers several advantages compared to human studies as purified diets can be used to meet several important conditions for diet comparisons. This includes equation of energy and protein intake irrespective of carbohydrate and fat content of the diets as well as elimination of effects of macronutrient sources (which are the same only differ in quantities) during comparisons of low-fat and low-carbohydrate diet archetypes. Thus, we studied C57BL/6J mice fed purified diets to test the hypothesis that (1) carbohydrate to fat ratio in the diets does not affect changes in body composition and metabolic health of obese mice under conditions of isocaloric energy restriction and equated protein, and (2) high dietary protein content of the diet has a beneficial effect on these health outcomes.

## 2. Materials and Methods

### 2.1. Animals and Experimental Design

The study was carried out at the animal research facilities of the Lithuanian Sports University. The males of C57BL/6J mouse strain were used in the experiment with all procedures approved by the Lithuanian State Food and Veterinary Service in 2018 (Ref. G2-90). The housing conditions of mice were set as follows: 20–21 °C ambient temperature, 40–60% humidity and an alternating 12-h light/dark cycle. Weaned mice were housed up to five animals per cage and fed *ad libitum* using a regular grain-based rodent chow diet (56.7 kcal% carbohydrate, 29.8 kcal% protein, 13.4 kcal% fat; Joniskio grudai, Lithuania) and had unrestricted access to a tap water. At 10 weeks of age mice were switched to the obesogenic high-fat and high-sugar diet (45% and 17.5% kcal from fat and sugar; D12451, Research Diets, New Brunswick, NJ, USA) for 18 weeks. At 20 weeks of age after 10 weeks of obesogenic feeding mice were moved into separate cages and measurements of food intake were carried out during the last three weeks of 8-week period before the start of caloric restriction (CR). Average daily energy intake of mice per gram of body mass was estimated based on food consumption of each mouse as assessed each week for three weeks by subtracting food leftovers from the initially provided food with corrections for effects of humidity on the pellet weight. The three-week average daily energy intake of the whole mouse colony was 0.41 ± 0.04 kcal/g/d. Afterwards a 6-week CR was applied while ten mice continued to be fed the obesogenic diet and served as age-matched controls for mice exposed to CR.

### 2.2. Caloric Restriction

28-week-old obese mice were randomly assigned to one of three weight-matched CR groups (*n* = 16 each) with different macronutrient distribution of diets (%kcal from fat, carbohydrate, protein) as provided by a supplier (Table 1): (1) low-fat diet, LFD (%kcal 20, 60, 20); (2) low-carbohydrate diet, LCD (%kcal 60, 20, 20) or (3) high-protein diet, HPD (%kcal 30, 35, 35). During 6-week CR was gradually increased from 20% (1st week) to 30% (2–4th week) and 40% (5–6th week) of daily energy intake by calculating the daily food ration for each mouse after adjustment for caloric density of the diets (4.1, 5.2 and 4.3 kcal/g for LFD, LCD and HPD, respectively). For CR phase food and energy intake was calculated for each mouse using the abovementioned colony’s daily energy intake reduced by the extent of caloric deficit and multiplied by actual data on body mass prior to CR. Thus, similar weighing mice received the same calorie content independently of the CR diet. Mice were fed each day at 8 AM.

### 2.3. Glucose Tolerance

Glucose tolerance tests were carried out after overnight fasting during the sixth week of CR as described previously [20]. Briefly, blood glucose concentrations were measured in blood samples from the tail vein using glucometer (Glucocard X-mini plus GT-1960, Arkray, Japan) in awake mice before glucose injection (0 min) and following intraperitoneal injection of glucose solution (2 g glucose per kg of body weight) 15, 30, 60, 90 and 120 min after injection. These measurements were used to estimate blood glucose area under curve (AUC) applying Prism 6.0 software (GraphPad Software Inc., San Diego, CA, USA).

### 2.4. Energy Metabolism and Physical Activity

Metabolic cage (OxyletPro, Panlab Harvard Apparatus, Barcelona, Spain) was used to assess respiratory quotient, energy expenditure and motor activity of freely moving mice [20]. After an overnight fast each mouse was weighed (ABS 80-4, Kern, Balingen, Germany) and housed in metabolic cage of a standard size for 3-h measurements with *ad libitum* access to water and without food. Metabolic cage was connected to the gas analyser (LE405, Panlab Harvard Apparatus, Barcelona, Spain) and the switching device (LE400, Panlab Harvard Apparatus, Barcelona, Spain) for control of the air flow set to 250 mL/min and 3-min switching time between measurements of O_2_ and CO_2_ concentrations in the metabolic cage and the external environment. After 1 h of acclimation in the metabolic cage, energy expenditure and respiratory quotient were calculated from 2-h averages using a software provided with the system (Metabolism v. 1.2, Panlab Harvard Apparatus, Barcelona, Spain). The integral of ground reaction forces of mice was used as a measure of physical activ-ity by using strain gauges mounted on the supporting constructions of the cage and rearings assessed as lifts of the mouse body above infrared beams of light set at 10 cm height. After the measurements the mouse was weighed again and transferred back to the home cage for continuation of the experiment.

### 2.5. Blood Lipids

At the end of the experiment mice were euthanized using CO_2_. Immediately following sacrifice blood lipids were measured from a 50 µL whole blood samples collected by cardiac puncture using a spectrophotometer (Cardiocheck PA, Polymer Technology Systems Inc., Whitestown, IN, USA) and test strips for lipids including total cholesterol (CHOL), high density lipoprotein (HDL) and triacylglycerol (TAG) (Ref. #1710, Polymer Technology Systems Inc., Whitestown, IN, USA). 

### 2.6. Body Composition

Throughout a period of CR mice were weighed with a precision of 0.1 g (440-45N, Kern, Balingen, Germany). After sacrifice heart, liver, skeletal muscles and body fat were removed and weighed with a precision of 0.1 mg (ABS 80-4, Kern, Balingen, Germany). Gastrocnemius, plantaris, soleus, tibialis anterior and extensor digitorum longus (EDL) muscles of both legs were removed, trimmed from all visible tendons and blotted dry before weighing. The average weights of these two muscles were used for analysis. The summed mass of all the muscles is referred to as the combined hindlimb muscle mass. Combined body fat mass was calculated as the sum of the subcutaneous, gonadal, mesenteric and perirenal fat which represented white adipose tissue and intrascapular fat representing brown adipose tissue as in previous studies [21,22].

### 2.7. Statistical Analysis

All data are presented as means with plotted individual data points unless otherwise stated. SPSS Statistics v20 (IBM, NY, USA) and Prism 6.0 software was used for the statistical analysis. Data normality was verified using Shapiro-Wilk test. Normally distributed data were analyzed with one-way analysis of variance (ANOVA) with Bonferroni’s *post hoc* test to assess differences between the diet groups. Non-parametric Kruskal–Wallis test with Dunn’s *post hoc* analysis was applied when means were not distributed normally. Two-way repeated measures ANOVA was used for analysis of changes in body mass and glucose levels during a glucose tolerance test as these measurements were performed repeatedly on the same animals. Analysis of covariance (ANCOVA) was applied using linear models to assess effects of mouse diet groups on energy expenditure where body mass and physical activity were used as covariates [23]. Linear regression analysis was also used and Pearson’s correlation coefficient was calculated to assess strength of the association between the variables. The level of significance was set at a *p*-value less than 0.05.

## 3. Results

### 3.1. All Diets Had Similar Effect on Body Composition in Obese Mice

**Figure 1:**
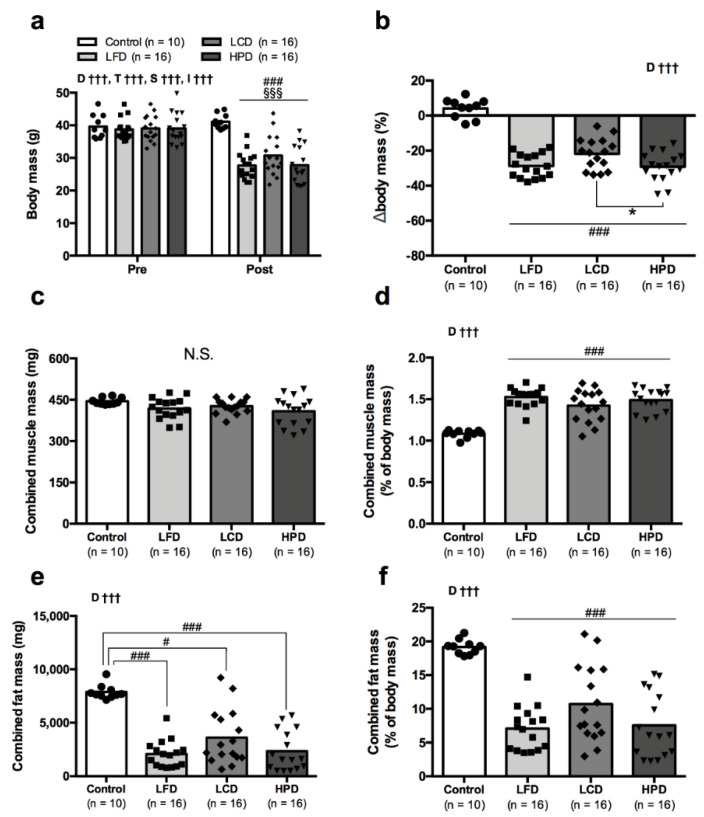
Changes in body mass (**a**,**b**), hindlimb muscle mass (**c**,**d**) and combined body fat (**e**,**f**) during a 6-week caloric restriction on low-fat (LFD), low-carbohydrate (LCD), high-protein (HPD) diets compared to the age-matched obese controls. Data are means with each dot represented by a single mouse. Statistical analysis: (**a**) Two-way repeated measures ANOVA with Bonferroni’s *post hoc* analysis was performed for effects of diet group and time, respectively; (**b**–**f**) One-way ANOVA with Bonferroni’s *post hoc* analysis was performed for diet group effect. N.S. indicates no statistical significance between compared means. ^†††^ *p* < 0.001 for effects of diet group (D), time (T), subject (S) and interaction (I). * *p* < 0.05 between diet groups connected by line. ^§§§^ *p* < 0.001 pre vs. post values. ^#^ *p* < 0.05, ^###^ *p* < 0.001 vs. obese controls.

Data on body composition is presented in Figure 1. CR induced substantial (*p* < 0.001) weight losses in mice on all three diets (Figure 1a). Body mass decreased by 28.7 ± 6.7, 21.8 ± 8.8 and 29.2 ± 8.3% for LFD, LCD and HPD, respectively (Figure 1b), and HPD led to a greater loss of body mass than LCD (*p* = 0.049). Body mass loss was not due to changes in the skeletal muscle mass which was not affected by the diets (Figure 1c). CR was associated with increase (*p* < 0.001) in relative muscle mass expressed as percentage of body mass for three diet groups (Figure 1d). In contrast to muscle mass, the combined fat mass was greatly reduced (*p* < 0.02–0.001) in all CR groups compared to the age-matched obese controls (Figure 1e). CR-induced decline in body fat was also significant (*p* < 0.001) for all three diets when fat mass was expressed as percentage of body mass (Figure 1f).

Weights of isolated hindlimb muscles, fat from different sites as well as liver and heart masses are presented in Table 2. CR had little impact on the skeletal muscles as judged by comparison to the age-matched obese controls, and only HPD group had a slightly lower mass of gastrocnemius (*p* = 0.041) and EDL (*p* = 0.034) compared to the controls. However, CR resulted in a substantial decrease in fat mass from all sites independently of the diet. LCD group tended to demonstrate smaller decreases in fat mass compared to the other two diet groups albeit with no significant differences. CR induced a decrease (*p* < 0.001) in liver mass for all diet groups. However, LFD and HPD groups preserved a body mass normalized liver mass whereas it was slightly reduced (*p* = 0.035) in LCD compared to the obese controls. CR led to a small decrease in heart mass for LFD (*p* = 0.006) and HPD (*p* = 0.0197), but not LCD compared to the obese controls. Relative heart mass expressed as percentage of body mass markedly increased (*p* < 0.01–0.001) with all diets compared to the control group.

### 3.2. Blood Glucose and Cholesterol Improved with Weight Loss

**Figure 2:**
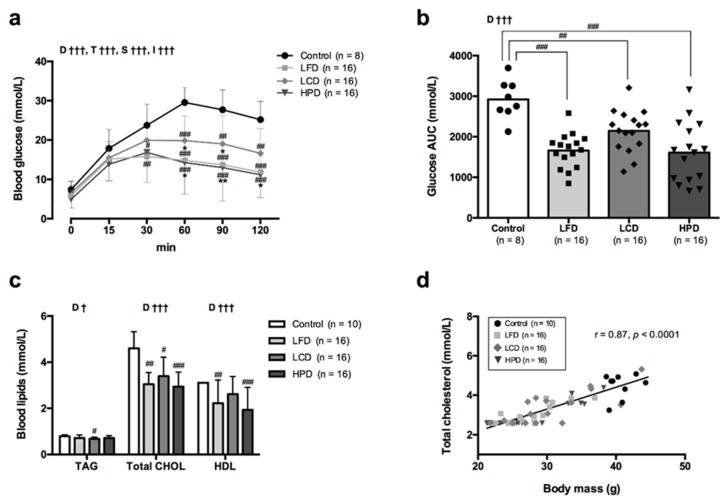
Blood glucose kinetics (**a**), blood glucose area under the curve (AUC) (**b**), blood lipid levels (**c**) and correlation between total blood cholesterol and body mass (**d**) following a 6-week caloric restriction on low-fat (LFD), low-carbohydrate (LCD), high-protein (HPD) compared to the age-matched obese controls. Abbreviations for (**c**): TAG, triacylglycerol; CHOL, cholesterol; HDL, high density lipoprotein. Data are means ± SD (**a**,**c**) or means with plotted individual values (**b**). Statistical analysis: (**a**) Two-way repeated measures ANOVA with Bonferroni’s *post hoc* analysis was performed for effects of diet group and time, respectively; (**b**,**c**^TAG^) One-way ANOVA with Bonferroni’s *post hoc* analysis was performed for diet group effect; (**c**^CHOL,HDL^) Non-parametric Kruskal-Wallis with Dunn’s *post hoc* analysis was performed for diet group effect. ^†^ *p* < 0.05, ^†††^ *p* < 0.001 for effects of diet group (D), time (T), subject (S) and interaction (I). ^#^ *p* < 0.05, ^##^ *p* < 0.01, ^###^ *p* < 0.001 vs. obese controls. * *p* < 0.05, ** *p* < 0.01 vs. LCD group.

Data on blood glucose and lipids is presented in Figure 2. CR induced significant improvements in glucose tolerance compared to the control group (Figure 2a). Glucose AUC was lower for all three diets compared to the control group though LCD led to higher glucose AUC compared to LFD and HPD (Figure 2b). Indeed, LCD group showed higher blood glucose levels at 60- and 90-min of the test compared to LFD and HPD groups (Figure 2a).

Blood TAG levels decreased (*p* = 0.032) only in the LCD group compared to the control group (Figure 2c), while total CHOL was markedly reduced in all three diet groups (Figure 2c). HDL cholesterol decreased for LFD (*p* = 0.0046) and HPD (*p* = 0.008). All mice in the control group had HDL cholesterol values equal or above measurable upper range (≥3.11 mmol/L), thus differences compared to the diet groups could be even higher. Also, this might explain why a tendency for reduction in HDL cholesterol for LCD group did not reach significance. The linear regression analysis revealed that total CHOL levels was strongly associated (r = 0.87, *p* < 0.0001) with body mass. Mice with lowest body mass usually had the lowest blood levels of total CHOL and vice versa (Figure 2d).

### 3.3. Energy Intake, Energy Metabolism and Activity Were Similar between the Diet Groups

**Figure 3:**
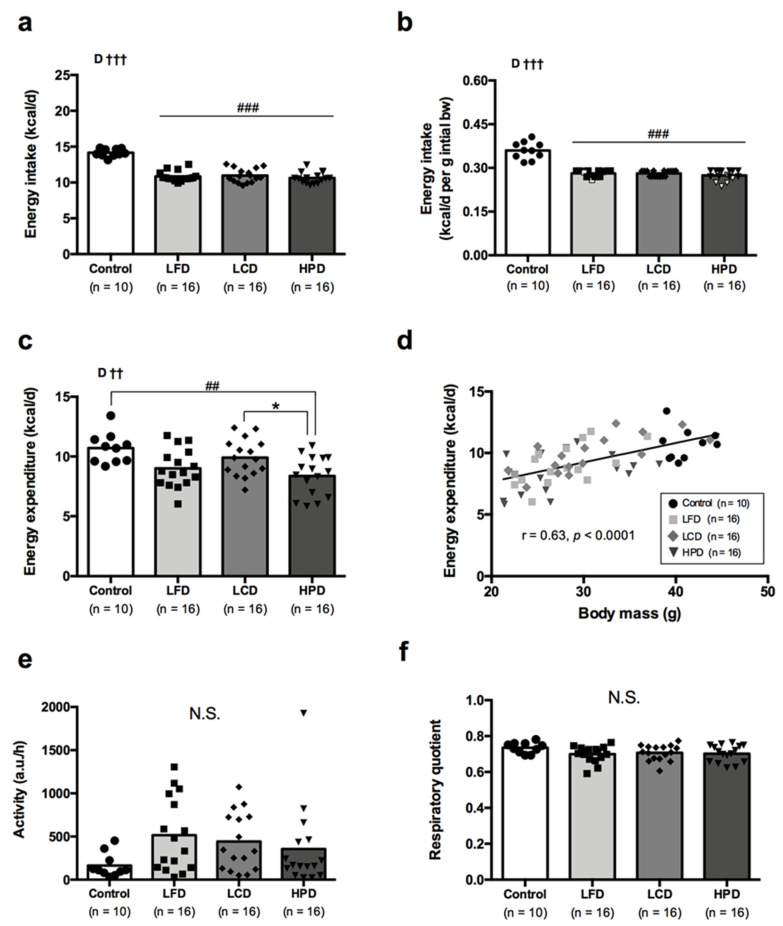
Energy intake (**a**,**b**), energy expenditure (**c**,**d**), physical activity level (**e**) and respiratory quotient (**f**) following a 6-week caloric restriction on low-fat (LFD), low-carbohydrate (LCD), high-protein (HPD) diets compared to the age-matched obese controls. Data are means with each dot represents a single mouse (white dots in Figure b indicate mice which had some food leftovers after the final day of CR). Statistical analysis: (**a**,**c**,**f**) One-way ANOVA with Bonferroni’s *post hoc* analysis was performed for diet group effect; (**b**,**e**) Non-parametric Kruskal-Wallis with Dunn’s *post hoc* analysis was performed for diet group effect. N.S. indicates no statistical significance between compared means. ^††^ *p* < 0.01, ^†††^ *p* < 0.001 for effect of diet group (D). ^##^ *p* < 0.01, ^###^ *p* < 0.001 vs. obese controls. * *p* < 0.05 vs. LCD group.

Data on energy intake and metabolism are presented in Figure 3. During CR energy intake did not differ between the diet groups and was lower by ~25% than for the obese controls (Figure 3a). Similarly, energy intake normalized to initial body mass before CR differed little between the diets and was on average ~23% lower compared to the controls (Figure 3b). It is worth mentioning that five mice in the HPD and two mice in the LFD group did not consume all prescribed food during CR and left some food in the feeders (indicated as white dots in Figure 3b). Energy expenditure did not differ between the groups of mice with exception of HPD which had lower energy expenditure than obese controls (*p* = 0.0027) and LCD group (*p* = 0.049) (Figure 3c). It might be associated with slightly greater body mass reduction in the HPD group as ANCOVA analysis with body mass and physical activity as covariates showed that body mass has strong effect (*p* < 0.0001) on energy expenditure irrespective of the diet. Moreover, a strong positive linear relationship between body mass and energy expenditure was also observed (r = 0.63, *p* < 0.001) (Figure 3d). There was a large intragroup variation in physical activity levels but without differences between the groups (Figure 3e). All CR groups showed a tendency of increased activity compared to the obese controls. Respiratory quotient did not differ between the groups of mice (Figure 3f).

## 4. Discussion

The main aim of our study was to investigate how macronutrient distribution in diet affects body composition and health markers in obese mice subjected to 6-weeks of caloric restriction. In contrast to our study the absolute majority of previous studies on diet comparisons used overfeeding interventions [24,25,26]. Our results showed that dietary carbohydrate and fat ratio in the hypocaloric and protein equated diets did not play a major role in weight loss. Also, we determined that isocaloric diet with higher protein content has similar effects on weight loss as low-fat or low-carbohydrate diets without any additional benefits on lean body mass retention. It appears that improvements in glucose tolerance and reduction in blood cholesterol were closely linked to weight loss rather than macronutrient distribution in diet. On the other hand, plasma triacylglycerol levels tended to show a decrease in mice fed a low-carbohydrate diet, compared to low-fat or high-protein diets. It appears that high dietary fat content might induce dissociation between improvements in glucose metabolism and lipid metabolism during caloric restriction.

Results of our caloric restriction study contradict some key concepts of the carbohydrate-insulin model (CIM) of obesity which has been advocated by some [14] and criticized by others [15]. According to CIM, high-carbohydrate diets elevate blood insulin secretion and “trap” fat in the body by suppressing fatty acid oxidation as well as promoting lipogenesis in the adipose tissue [14]. It is speculated that high-carbohydrate diets lead to low availability of energy in metabolically active tissues and create a state of “internal starvation” that adaptively decrease energy expenditure and promotes energy intake, and thus weight gain. However, these predictions are supported by only a few studies [27] that have been criticized for poor methodology [28,29,30]. On the hand, there is a significant amount of evidence that contradicts CIM and shows no effect of reduced carbohydrate intake on weight loss compared to higher carbohydrate intake when energy and protein intake are controlled [31,32]. Gardner et al. [32] showed that baseline insulin secretion and genotype relevant to carbohydrate and fat metabolism were not associated with weight change using different diets. Hu et al. [24] have recently demonstrated that CIM does not explain the effects of dietary macronutrient composition on adiposity in mice. Our results also show no difference between low-carbohydrate and low-fat diets in fat loss and energy expenditure of mice during caloric restriction with equated protein intake.

It appears that the observed benefits of low-carbohydrate diets on weight loss in less rigorously controlled studies might be simply attributable to reduced overall energy and increased protein intake rather than diet *per se*. Reduction in dietary carbohydrate content is often compensated for by other macronutrients such as dietary protein. Soenen et al. [33] showed that “high-protein” component is more important than “low-carbohydrate” component on weight loss and subsequent weight maintenance in humans. Dietary protein has a greater positive effect on thermogenesis [7], satiety [6] and lean body mass [34,35,36] compared to dietary carbohydrates or fat. Evidence shows that higher protein intake might be beneficial for weight management [8,9,33]. However, our study showed no significant benefits for health and changes in body composition when protein intake was increased from 20% to 35% kcal. It appears that 20% kcal from of protein in the diet might be enough to maintain muscle mass in obese mice during caloric restriction. Nevertheless, we did observe a tendency for a larger decrease in body mass and energy expenditure for the high-protein diet compared to low-carbohydrate diet. Indeed, five mice out of sixteen mice did not consume all the prescribed food in the high-protein group while only two mice behaved in this way in the low-fat group and none in the low-carbohydrate group. A small reduction in food intake might induce a slightly greater energy deficit which over time could cause more pronounced adaptive thermogenesis in mice on high-protein diet compared to the other two diets [37]. This, together with slightly greater weight loss in those mice might suppress basal metabolic rate and did not translate into additional health benefits compared to the other two diets in our study.

Studies of diet-associated weight loss in humans suggest that approximately 25% of weight loss is due to decrease in lean body mass which is likely to be associated with loss of muscle mass [36]. Interestingly, there was no decrease in hindlimb muscle mass of obese mice in our study and muscle-to-body mass ratio increased significantly after caloric restriction using all three diets. There was also a relatively small reduction in heart mass which resulted in increased heart-to-body mass ratio. Heart is one of the most protected organs in mice during caloric restriction [38]. In contrast to heart, liver mass showed significant decrease but liver-to-body mass ratio was maintained which might indicate functional adaptation due to reduction in energy intake, liver glycogen and metabolism properties during caloric restriction, though analysing factors that come into play in the preservation of various organs’ mass was beyond the scope of our current investigation. 

We have examined effects of caloric restriction on metabolic health in greater detail. All three diets promoted improvements in glucose tolerance during caloric restriction compared to obese controls, but low-carbohydrate diet tended to have a smaller effect compared to the other diets. Impaired glucose tolerance with low-carbohydrate and/or ketogenic diets was linked to reduced α- and β-cell mass leading to decrease in insulin synthesis and secretion in rodents [39,40,41,42,43,44]. Humans adapted to low-carb-high-fat diet had lower skeletal muscle GLUT4 and IRS1 contents which was linked to reduced their glucose tolerance [45]. There was also a reduction in Glut4 gene expression levels in mice exposed to a ketogenic diet [46]. It might be expected that diets higher in carbohydrate content are better suited to promote glucose disposal and glucose tolerance. Nevertheless, our results show that caloric restriction-induced loss of body fat rather than macronutrient composition of diet is a major factor in improvements of glucose control [20,47,48,49]. Cholesterol reduction was also strongly correlated with weight loss and was greatest with the low-fat and high-protein diets in our study. On the other hand, plasma triacylglycerol levels were significantly reduced with the low-carbohydrate diet only. Low triacylglycerol seems to be a common feature of the low-carbohydrate diets in humans as well [50,51,52]. Elevated triacylglycerol and decreased HDL-cholesterol levels are often associated with cardiovascular diseases [53,54]. Whether the low triacylglycerol and adequate HDL-cholesterol levels could mitigate the risks of elevated LDL-cholesterol attributable to the low-carb diets is under the intensive debate at the moment and more research is needed to elucidate this dispute [55].

Results of our study should be interpreted with caution in projecting long-term benefits to metabolic health. Effects on multiple metabolic indicators still needed to be investigated to comprehensively reveal dietary macronutrient role in metabolic health. We focused on short-term weight management in markedly obese mice using a contrasting macronutrient distribution in a state of caloric restriction. Rationale regarding our dietary choices such as a high-protein diet for body composition management might contradict with evidences from lifespan extension studies in rodents. Caloric restriction with accompanying protein and/or BCAA restriction is often suggested for promoting metabolic health during aging [56,57] although there are studies indicating that caloric restriction *per se* is the dominant factor in those improvements [58]. However, this topic was beyond the scope of our investigation and future studies are required to fulfil gaps of what really constitutes a metabolically healthiest diet.

## 5. Conclusions

In summary, our results show that macronutrient distribution in diet has a rather small effect on changes in body composition and glucose tolerance of obese mice subjected to isocaloric energy restriction. However, blood lipid profile seems to be affected by the carbohydrate and fat ratio in the diet. Increase in protein intake from 20% to 35% of total calories did not provide additional benefits to body composition and health markers. Thus, the primary emphasis in order to improve health in obesity should be placed on overall energy intake rather than dietary macronutrient distribution.

## Figures and Tables

**Table 1:** Nutritional characteristics of the obesogenic control, low-fat (LFD), low-carbohydrate (LCD) and high-protein (HPD) diets used in the experiment.

	Control		LFD		LCD		HPD	
Energy status	Ad libitum		CR up to 40%		CR up to 40%		CR up to 40%	
Diet’s manufacturer	Research Diets Inc.		Research Diets Inc.		Research Diets Inc.		Research Diets Inc.	
Diet’s number	D12451		D17100401		D12492		D17100402	
Fat (kcal%)	45		20		60		30	
Carbohydrate (kcal%)	35		60		20		35	
Protein (kcal%)	20		20		20		35	
**Ingredients (in g and kcal)**								
***Protein:***								
Casein	23.3	93.3	20.0	80.1	25.8	103.3	38.2	152.6
L-cystine	0.35	1.4	0.30	1.2	0.35	1.4	0.53	2.1
***Carbohydrate:***								
Corn starch	8.5	33.9	40.5	162.1	0	0	16.1	64.4
Maltodextrin	11.7	46.6	12.5	50.0	16.1	64.6	15.9	63.6
Sucrose	20.1	80.6	6.9	27.5	8.9	35.5	7.3	29.1
***Fibre:***								
Cellulose	5.83	0	5.0	0	6.5	0	5.3	0
***Fat:***								
Soybean oil	2.9	26.2	2.5	22.5	3.2	29.1	2.65	23.8
Lard	20.7	186.3	6.5	58.5	31.6	284.8	11.66	104.9
***Minerals:***								
Mineral mix S10026	1.17	0	1.00	0	1.29	0	1.06	0
DiCalcium phosphate	1.52	0	1.30	0	1.68	0	1.38	0
Calcium carbonate	0.64	0	0.55	0	0.71	0	0.58	0
Potassium citrate	1.92	0	1.65	0	2.13	0	1.75	0
***Vitamins:***								
Vitamin mix V10001	1.17	4.7	1.00	4.0	1.29	5.2	1.06	4.2
Choline bitartrate	0.23	0	0.20	0	0.26	0	0.21	0
**Total**	100	473	100	406	100	524	100	430
**Kcal/g**	4.7		4.1		5.2		4.3	

**Table 2:** Weights of isolated hindlimb muscles, fat from different sampling sites and liver as well as heart after a 6-week caloric restriction on low-fat (LFD), low-carbohydrate (LCD) or high-pro (HPD) diets compared to the age-matched control group on obesogenic feeding.

	Control (*n* = 10)	LFD (*n* = 16)	LCD (*n* = 16)	HPD (*n* = 16)
**Hindlimb muscles:** *(in miligrams)*				
Gastrocnemius	135.8 ± 3.2	125.4 ± 12.8	129.2 ± 8.4	121.6 ± 18.0 ^#^
Plantaris	17.7 ± 0.6	16.4 ± 1.9	16.9 ± 1.3	16.2 ± 2.2
Soleus	9.7 ± 0.6	8.8 ± 0.8	9.1 ± 0.9	9.0 ± 1.1
Tibialis anterior	47.9 ± 2.9	48.2 ± 3.3	47.9 ± 2.8	47.2 ± 4.9
EDL	11.3 ± 0.6	10.4 ± 1.2	10.6 ± 0.6	10.1 ± 1.4 ^#^
**Major fat sites:** *(in miligrams)*				
Subcutaneous	3286.6 ± 405.4	880.1 ± 591.8 ^###^	1595.1 ± 1210.0 ^#^	1036.7 ± 894.8 ^###^
Gonadal	2275.3 ± 303.1	549.1 ± 364.9 ^###^	1047.7 ± 714.2 ^###^	617.4 ± 547.4 ^###^
Mesenteric	1152.7 ± 207.0	357.6 ± 162.5 ^###^	511.8 ± 349.6 ^##^	381.6 ± 207.2 ^###^
Perirenal	1007.3 ± 166.5	174.5 ± 147.9 ^###^	348.6 ± 314.1 ^##^	207.1 ± 203.1 ^###^
Intrascapular brown	159.4 ± 31.2	110.7 ± 24.0 ^###^	107.8 ± 27.8 ^###^	106.1 ± 28.6 ^###^
**Organs:** *(in miligrams)*				
Liver	1330.0 ± 304.0	848.5 ± 249.5 ^###^	847.2 ± 171.8 ^###^	876.2 ± 208.2 ^###^
Heart	142.7 ± 10.1	130.3 ± 7.5 ^##^	134.1 ± 9.2	131.8 ± 8.9 ^#^
*(% of body mass)*				
Liver	3.22 ± 0.61	3.03 ± 0.50	2.76 ± 0.20 ^#^	3.14 ± 0.25
Heart	0.35 ± 0.02	0.48 ± 0.06 ^###^	0.45 ± 0.08 ^##^	0.49 ± 0.09 ^###^

Means ± SD. ^#^ *p* < 0.05, ^##^ *p* < 0.01, ^###^ *p* < 0.001 vs. obese controls.

## Data Availability

The data that support the findings of this study are available from the corresponding author upon reasonable request.
